# DMSO-Induced Unfolding of the Antifungal Disulfide Protein PAF and Its Inactive Variant: A Combined NMR and DSC Study

**DOI:** 10.3390/ijms24021208

**Published:** 2023-01-07

**Authors:** András Czajlik, Ágnes Batta, Kinga Kerner, Ádám Fizil, Dorottya Hajdu, Mária Raics, Katalin E. Kövér, Gyula Batta

**Affiliations:** 1 Department of Organic Chemistry, Faculty of Science and Technology, University of Debrecen, Egyetem tér 1, H-4032 Debrecen, Hungary; 2 Department of Biochemistry, Institute of Biochemistry and Molecular Biology, Semmelweis University, Tűzoltó utca 37-47, H-1094 Budapest, Hungary; 3 Department of Biophysics and Cell Biology, Faculty of Medicine, University of Debrecen, Egyetem tér 1, H-4032 Debrecen, Hungary; 4 Department of Inorganic and Analytical Chemistry, Faculty of Science and Technology, University of Debrecen, Egyetem tér 1, H-4032 Debrecen, Hungary; 5 MTA-DE Molecular Recognition and Interaction Research Group, Department of Inorganic and Analytical Chemistry, Faculty of Science and Technology, University of Debrecen, Egyetem tér 1, H-4032 Debrecen, Hungary

**Keywords:** antifungal protein, PAF, disulfide bond, DMSO-induced unfolding, nuclear magnetic resonance (NMR), differential scanning calorimetry (DSC)

## Abstract

PAF and related antifungal proteins are promising antimicrobial agents. They have highly stable folds around room temperature due to the presence of 3–4 disulfide bonds. However, unfolded states persist and contribute to the thermal equilibrium in aqueous solution, and low-populated states might influence their biological impact. To explore such equilibria during dimethyl sulfoxide (DMSO)-induced chemical unfolding, we studied PAF and its inactive variant PAF^D19S^ using nuclear magnetic resonance (NMR) and differential scanning calorimetry (DSC). According to the NMR monitoring at 310 K, the folded structures disappear above 80 *v*/*v*% DMSO concentration, while the unfolding is completely reversible. Evaluation of a few resolved peaks from viscosity-compensated ^15^N-^1^H HSQC spectra of PAF yielded ∆G = 23 ± 7 kJ/M as the average value for NMR unfolding enthalpy. The NMR-based structures of PAF and the mutant in 50 *v*/*v*% DMSO/H_2_O mixtures were more similar in the mixed solvents then they were in water. The ^15^N NMR relaxation dynamics in the same mixtures verified the rigid backbones of the NMR-visible fractions of the proteins; still, enhanced dynamics around the termini and some loops were observed. DSC monitoring of the T_m_ melting point showed parabolic dependence on the DMSO molar fraction and suggested that PAF is more stable than the inactive PAF^D19S^. The DSC experiments were irreversible due to the applied broad temperature range, but still suggestive of the endothermic unfolding of PAF.

## 1. Introduction

Approximately one billion people suffer from fungal diseases, which due to increasing resistance cause around 1.5 million deaths per year [1]. Fungal infections also affect agriculture, food supply, wildlife, and even cultural heritage. Therefore, the discovery and understanding of the mode of action of new antifungal agents [2,3,4,5,6] are urgent and important. The small, cysteine-rich, and cationic proteins secreted by the filamentous ascomycetes and other organisms are among the most promising new antifungal drug candidates [7]. They have proven to be highly efficient against different human [8,9] and plant [10,11] pathogenic fungi and do not show cytotoxic effects on mammalian cells either in vitro [12] or in vivo [13,14]. These “mini proteins” (MPs) adopt characteristic β-barrel folds [15,16,17] stabilized by three or four disulfide bonds. They are stable in a broad temperature and pH range [16] and resistant against proteolytic degradation. However, all details of their antifungal effects are still not known and deserve further studies. Interestingly, their mode of action is quite different even for proteins of high homology. For example, NFAP [18] and NFAP2 [19,20] disrupt the cell wall and, thus, induce apoptosis [21,22,23]. In contrast, PAF penetrates the cell membrane without membrane destruction [24,25]. It localizes in the cytoplasm and triggers the generation of reactive oxygen species (ROS) [24], which ultimately cause the death of the cells. The mode of action is connected to cell signaling processes, and other proteins are linked to the mechanism, including heterotrimeric G-proteins [26] and protein kinase A [27]. Cation channels and glucosylceramide synthesis may also play a role. The γ-core motif of the PAF sequence is necessary for the antifungal effect. This was not a surprise, since such motifs with a sequence of either GXCX_3-9_C (dextromeric form) or CX_3-9_CXGX_1-3_/CX_3-9_GXCX_1-3_ (levomeric forms) are common in antimicrobial disulfide proteins. 

The three-dimensional structure and dynamical properties of PAF have been described previously [16,28]. Most related proteins adopt β-barrel folds; however, it was found in the case of PAF that a significant population might be in a dynamic conformational equilibrium at room temperature. Hidden conformers may play a role (e.g., conformational selection) in the antifungal effect; therefore, further investigations are needed. In order to learn more about the unfolded states, chemical unfolding experiments were carried out with PAF and its biologically inactive mutant PAF^D19S^.

For this purpose, DMSO was chosen, which often helps to dissolve compounds that are insoluble in water and, therefore, is important in protein–drug interaction studies of pharmaceutical interest. In this work, the DMSO-induced unfolding was followed by NMR spectroscopy and differential scanning calorimetry to yield the thermodynamics of the unfolding. We thought that comparing the unfolding of PAF and the inactive PAF^D19S^ variant using two independent physicochemical methods could be interesting. Generally, both methods are widely accepted for protein structure (NMR) and stability (DSC) measurements. The NMR experiments are carried out at constant temperatures (in the range of 298–320 K), with proven reversibility. In contrast, the DSC temperature scans at fixed DMSO concentrations cover a ca. 100 K temperature range with a high-temperature end at 400 K. DSC displays the protein phase transition when the folded and unfolded populations are the same (50–50%), which is reflected in the T_m_ melting point value. However, DSC experiments cause irreversible unfolding because of the high end temperature. 

The amount of folded protein fraction was measured by NMR as a function of the DMSO concentration. In the end, the three-dimensional structures and dynamics of PAF and PAF^D19S^ were characterized in the presence of 50% DMSO; however, we obtained no information about the NMR-invisible part of the protein conformational ensemble.

## 2. Results

### 2.1. PAF and PAF^D19S^ Have Similar Solution Structures in Water and in a 50% DMSO–Water Mixture

**Figure 1:**
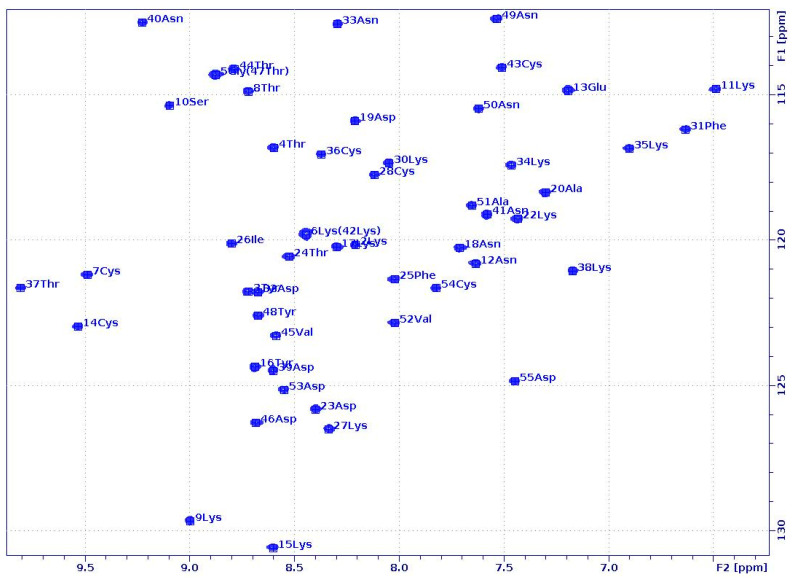
The assigned ^1^H-^15^N HSQC spectrum of the PAF in the presence of 50% DMSO, added to an acetate buffer (pH 4.5), as measured with a NEO 700 MHz spectrometer (Bruker) at 310 K temperature. Residue specific NH peak assignments are labelled: see also BMRB 34655 deposition in case of overlap.

Solution NMR spectroscopy was employed to determine the structure and dynamics of PAF and PAF^D19S^ in 50 *v*/*v*% water/DMSO-d_6_ solvent mixtures. At this DMSO level, the protein NMR spectra were clearly affected, and to some extent (~13% of the population) the PAF started to unfold (vide infra, Figure 1). Nevertheless, the visible and observed NMR signals still allowed structure and dynamics studies. Our main goal was to investigate the local and global conformational changes in the proteins caused by DMSO. Since all amide NH-s and Asn side-chain NH_2_ groups were well resolved in the ^1^H-^15^N HSQC spectra (Figure 1), this suggests that the NMR-visible fractions of both proteins have folded structures in the presence of 50% DMSO. The level of the ^1^H- and ^15^N-assignments proved to be acceptable for both PAF (86 and 81%, respectively) and PAF^D19S^ (81 and 81%, respectively). Furthermore, almost all of the backbone ^1^H and ^15^N atoms were identified in both proteins.

**Figure 2:**
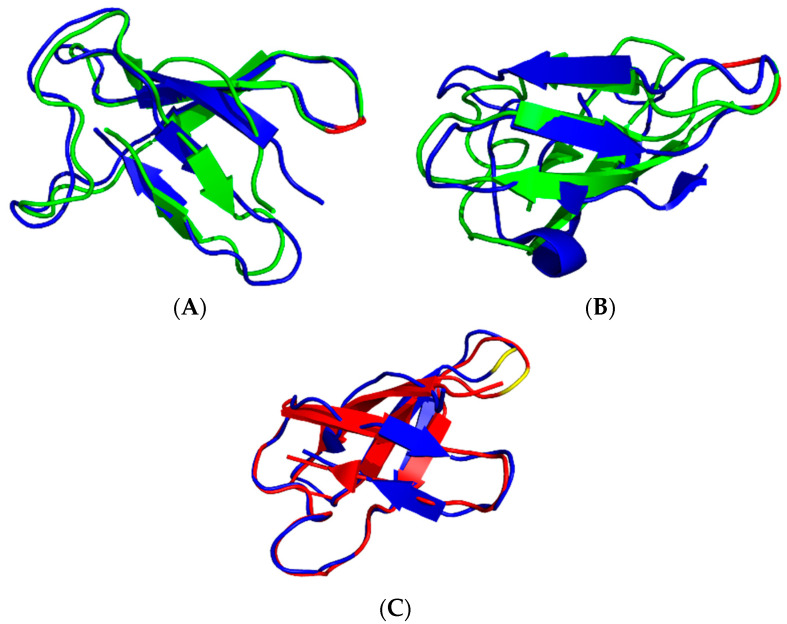
Comparison of the solution backbone structures in water (blue) and in 50% DMSO (green). PAF (2MHV vs. 7PGD) (**A**) and PAF^D19S^ (2NB0 vs. 7NXI) (**B**). Overlaid structures of PAF (blue) and PAF^D19S^ (red) as measured in 50% DMSO (**C**). The mutation site is labelled with red (**A**,**B**) or yellow (**C**). (PyMOL visualization software was used).

In fact, most of the unassigned resonances belonged to NZ, HD, HE, or HZ of such lysine (Lys) side chains, which face outwards and do not interact with other amino acids. A good percentage of the NOEs proved to be medium- (13.4 and 13.8%) and long-range (34.2 and 34.5%), indicating β-barrel structures, similar to what we found in water [28]. The high number of distance restraints (17.38 and 17.42 NOEs/residue) collected from the spectra were almost comparable with those in the ^15^N-/^13^C-labelled PAF structure [28], and even higher than in the case of PAF^D19S^ [29]. In the end, we obtained good-quality structures with a relatively well-defined protein backbone for both PAF and PAF^D19S^ (PDB: 7PGD and 7NXI, BMRB: 34,655 and 34612, Figure 2). Although a lot of side chains are more flexible, the relatively low heavy-atom RMSD values of the final models (0.93 ± 0.09 and 0.85 ± 0.07 Å) suggest the stability of the folds.

Both proteins adopted β-barrel structures, characteristic of the PAF-cluster antifungal proteins, even in the presence of 50% DMSO. As usual, they consisted of two antiparallel β-sheets formed by five antiparallel β-strands. These β-sheets were the same in 50% DMSO as in aqueous solution, i.e., containing the β1-, β2-, β3 and β1-, β4-, β5-strands, respectively. Interestingly the N-terminal β1-strand was partially irregular in both proteins, which may have contributed to the lower stability of the proteins in 50% DMSO, as shown by differential scanning calorimetry (DSC). For PAF, the β-strands were located between Gly5 and Thr8 (β1), Glu13 and Lys17 (β2), Asp23 and Lys27 (β3), Cys43 and Val45 (β4), and Ala51 and Asp53 (β5). In the case of PAF^D19S^, their positions were very similar: Lys2-Thr8 (β1), Glu13-Tyr16 (β2), Thr24-Lys27 (β3), Thr44-Asp46 (β4), and Val52-Cys54 (β5). Furthermore, the four loop regions (one longer and three shorter) between the β-strands and the hidden central core formed by three disulfide bonds were also present. Finally, the aromatic rings of Tyr 3 and Tyr16 still interacted with one another in both proteins and may play some role in the stability of the N-terminal β-sheet.

The three-dimensional structure of PAF in 50% DMSO solution (Figure 2A) did not change much compared to that determined in water, as proven by the small RMSD value (1.455 Å) between the two structures (2MHV and 7PGD).

Still, the β1-strand, the fourth loop region, and the beginning of the β5-strand showed conformational differences in 50% DMSO. This was especially noticeable at the N-terminal, where the β1-strand was shorter and more flexible. In fact, the conformation around Lys2 seemed irregular, as shown by Ramachandran analysis in the PDB validation report. Furthermore, the first β-turn (Asp32-Asn33) of the two in the native structure had a less regular conformation in 50% DMSO. Some minor differences were also detected in other loop regions. 

As mentioned earlier, PAF^D19S^ forms a typical β-barrel fold both in water (2NB0) and in 50% DMSO (7NXI). All of the β-strands were present in the mixed solvent, although the first one was slightly irregular and shorter than in the native structure (Figure 2B). Here, the Lys2 showed an atypical conformation, similar to PAF in 50% DMSO. In the presence of DMSO, the 3D structure of PAF^D19S^ more closely resembled the conformation of PAF (RMSD: 1.376Å) (Figure 2C) than that of PAF^D19S^ in water (RMSD: 3.220Å). Compared to the native PAF^D19S^ structure in water, the loop regions were significantly different due to the impact of DMSO—especially the fourth and, to a lesser extent, the second and third loops—and they were more similar to those that were found in PAF with DMSO. The solvent-dependent changes in the fourth loop and the neighboring β-strands were obvious at the C-terminal part of the D19S variant. Furthermore, a β-turn (Asp32-Asn33) typical of PAF was also identified in 50% DMSO. Still, the 39–42 region remained twisted, although it did show a rather turn-like structure and fewer helical properties than observed in water. 

According to the NMR analysis, the general folding and the backbone conformation remained stable and mostly unchanged in the presence of 50% DMSO for both proteins. In contrast, the majority of the side chains showed conformational heterogeneity. The “abcabc” disulfide pattern was fixed during the calculations; however, the disulfide conformations were different within the NMR structural ensemble. Thus, the hydrophobic core, including the disulfides, became slightly more collapsed. It should be noted that NMR has known limitations for determining disulfide conformations [30] compared to X-ray [17].

**Figure 3:**
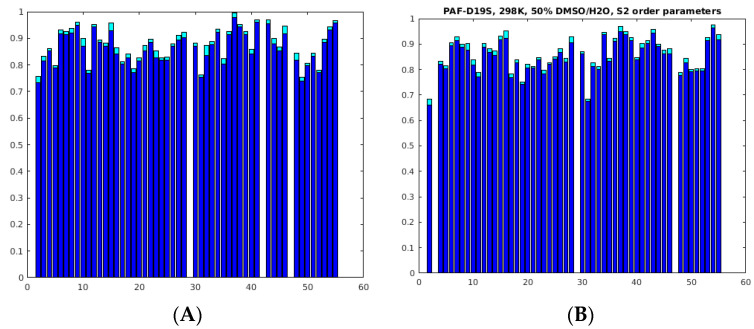
S^2^ order parameters of the NH vectors in PAF ((**A**), 310 K, 70.96 MHz) and PAF^D19S^, ((**B**), 298 K, 50.68 MHz), as measured from ^15^N relaxation experiments, and the Lipari–Szabo method was used for evaluation. Errors are shown on the top of the bars. A few data are not shown due to spectral overlap and from 29-Pro residue (see also Supplementary Table S1B).

To obtain further insight into the dynamical properties of our proteins in a 50% DMSO/water solvent mixture, ^15^N NMR (50.68 or 70.96 MHz) relaxation experiments (T_1_, T_2_ and ^15^N–(^1^H) hetNOE) [31] were performed at 298 K (PAF^D19S^) and at 310 and 320 K (PAF) temperatures. For data analysis, both the Lipari–Szabo model-free method [32] and the reduced spectral density mapping [33] approach were used. For PAF, the global correlation time at 310 K was more than twice as high (τ_c_ = 7.7 ± 0.3 ns) as in water at 304 K temperature (3.0 ns) [16]. The global correlation time for PAF was only 1.9 ± 0.6 ns at 320 K in H_2_O buffer, which was increased to 6.2 ± 0.3 ns by adding 50% DMSO. The same tendency was true for PAF^D19S^, and τ_c_ = 12.0 ± 0.3 ns was measured at 298 K with 50% DMSO as compared to the 3.0 ns value obtained in water. The huge increase in global correlation time was due to the high viscosity change caused by 50% DMSO at lower temperatures. Therefore, it is preferable to run the NMR relaxation experiments at higher temperatures if a significant amount of DMSO is present. The S^2^ order parameters in 50% DMSO were high for the majority of NH vectors, similar to the observations in water [16] (Figure 3). 

**Figure 4:**
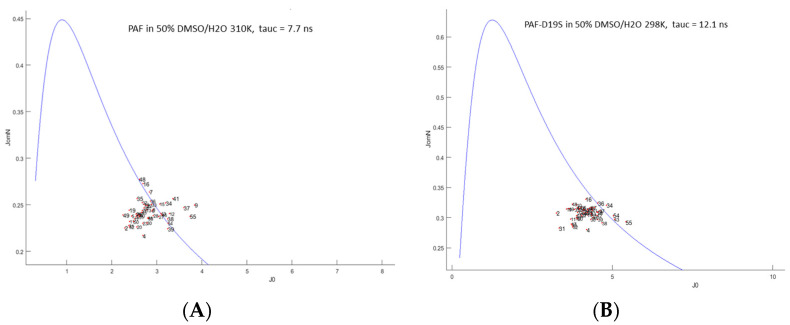
Reduced spectral density mapping as obtained in 50 *v*/*v*% DMSO from ^15^N relaxation data for PAF ((**A**), 310 K) and PAF^D19S^, ((**B**), 298 K). The same input experimental data were used as for Figure 3 (see also Supplementary Table S1). The continuous curve represents the absence of internal motion. Spectral densities (i.e., the strength of radiofrequency (RF) fields from molecular motions) at the ^15^N frequency JomN are shown as a function of RF fields close to zero frequency J0 (slow-motion regime). The units of both axes are 10^−9^ (s rad^−1^).

In the case of PAF, the S^2^ average was 0.87 ± 0.06 at 310 K, except for 2Lys, 11Lys, 19Asp, 31Phe, 49Asn, and 52Val with slightly lower values. The order parameter distribution was similar at 298 K for PAF^D19S^, i.e., S^2^ = 0.86 ± 0.07, and the 2Lys, 11Lys, 17Lys, 19Ser, 31Phe, and 48Tyr residues were slightly more mobile. Interestingly the C-terminal β-strand (β5) seemed to be less rigid if compared to other strands on the ps/ns timescale. The reduced spectral density mapping of PAF supports a rigid backbone, and only the 2Lys, 4Thr, Lys9, 11Lys, 31Phe, Thr37, 49Asn, 52Val, and Asp55 show slightly increased dynamics (Figure 4). 

The analysis of PAF^D19S^ gave a rather similar result; here, the outliers were 2Lys, 4Thr, 19Ser, 23Asp, 31Phe, 49Asn, 52Val, and Asp55. Moreover, plots of the R_1_ * R_2_ ^15^N-relaxation rates [34] (not shown) did not prove chemical exchange for any residue at the ps/ns timescale.

In summary, the observed NH order parameters of PAF and PAF^D19S^ in 50% DMSO verify dominantly rigid backbone structures similar to that observed in water, but the global correlation times increased significantly. Still, the small fluctuations in dynamics suggest that the unfolding process begins at the termini of both proteins, as expected [28].

Our NMR studies proved that the DMSO-induced unfolding of PAF is reversible around room temperature, and even the totally unfolded protein in 100% DMSO can be recovered upon dilution in water in the native form with untouched disulfide bonds (Figures S1 and S2). To follow the progress of unfolding, NMR- and DSC-monitored titrations were carried out starting with either 0 or 100% DMSO. NMR experiments in both directions showed the same unfolding/refolding process. Apparent unfolding started at ca. 40% DMSO, and the ^15^N-^1^H HSQC-NMR signals completely disappeared around 82 *v*/*v*% DMSO concentration, demonstrating that no folded PAF could be observed. In agreement with this, simple 1D ^1^H-NMR spectra showed extremely broad lines in the presence of more than 80% DMSO (Figures S1 and S2). According to the HSQC titration of PAF, nearly 10 M DMSO concentration (~70 *v/v*%) is required to unfold 50% of the proteins. It should be noted that, under extreme conditions (i.e., high temperature (90 °C) and adding DTT as a reducing agent), PAF unfolding becomes irreversible, resulting in the formation of inactive oligomers (Figure S4).

### 2.2. Unfolding Monitored by Differential Scanning Calorimetry (DSC)

**Figure 5:**
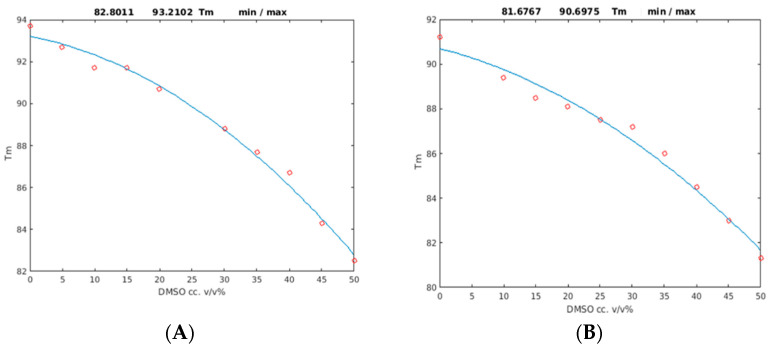
Thermodynamic stability of PAF (**A**) and PAF^D19S^ (**B**) as a function of the DMSO concentration. Second-order polynomials fitted to the melting point temperatures (T_m_) are shown in both cases. T_m_ values are shown in °C on the vertical scale.

DSC experiments were conducted in order to determine the stability differences between the two proteins that might be reflected in melting temperature (T_m_) changes. PAF and its mutant were irreversibly denatured in the DSC experiments due to the high temperatures (130 °C) at the end of the DSC heating protocol, and repeated DSC experiments showed no reproducible profiles. As expected, we found that the wild-type protein was more stable than the D19S mutant. The data show that while the T_m_ of the PAF was 93.2 ± 0.8 °C, it was only 90.7 ± 1.0 °C for PAF^D19S^ in the acetate buffer. Adding DMSO to the solutions gradually destabilized the proteins. The T_m_ values were significantly decreased at a 50% DMSO concentration, with 82.8 ± 0.7 °C for PAF and 81.7 ± 0.9 °C for the mutant. Moreover, this difference was smaller when compared to the T_m_ values in the absence of DMSO. The correlation between the T_m_ melting points and the DMSO concentration (0–50%) could be best fitted with parabolic functions (the R^2^ values were 0.99 for PAF and 0.98 for PAF^D19S^; Figure 5 and Table 1).

The T_m_ curves prove that the denaturation proceeded parallel with the DMSO concentration. Using the DSC technique, we detected some folded PAF even at 55% DMSO concentration, in contrast to PAF^D19S^, which became unstructured at the same DMSO content. At 62.5% DMSO, no folded structures were present in either protein. The DSC studies showed that the PAF was more stable than its biologically inactive variant. Its T_m_ melting point value was systematically higher in any DMSO/water solvent mixture, and the full unfolding took place at higher (62.5%) DMSO contents. The lower stability of PAF^D19S^ might be explained by a two-state unfolding as opposed to the three-state model found for PAF in temperature-induced unfolding studies [28]. However, it should be noted that temperature-induced and chemical unfolding are different physicochemical effects. 

### 2.3. Unfolding Monitored by NMR 

**Figure 6:**
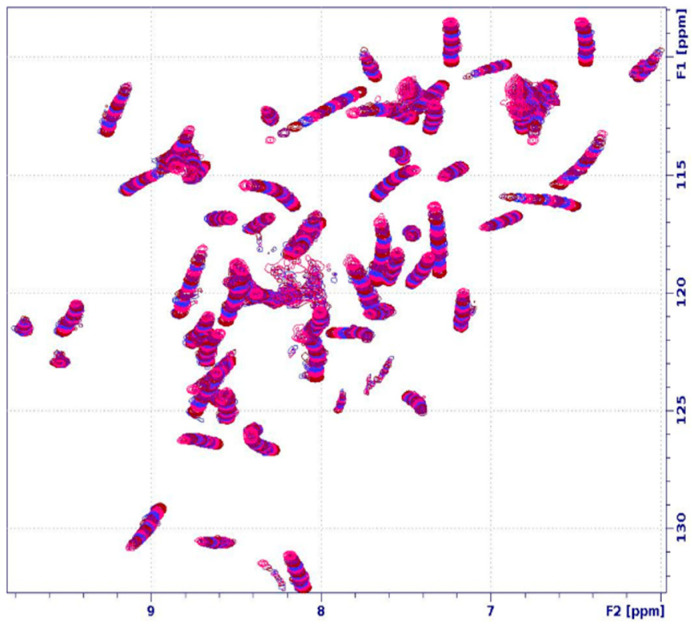
Titration of ^15^N-PAF with gradually increasing the DMSO-d_6_ concentration at 310 K temperature, as measured using a Bruker AVANCE-II 500 MHz spectrometer. The series of ^1^H-^15^N HSQC spectra display the changes in the chemical shifts and intensity of the amide NH groups.

The conformational selection model [35,36] of molecular recognition is believed to be a very efficient mechanism in biomolecular recognition. From this point of view, there is an interesting question as to whether “rigid” proteins (such as our cyclic disulfide proteins) may have such a conformational landscape that would allow this mechanism. To this end, we have previously shown [28] by CEST-NMR [37] that sporadic conformers exist in thermal equilibrium around room temperature and that their populations can be controlled by temperature. Here, we extended this method with chemical-induced unfolding in our model systems. A series of ^1^H-^15^N HSQC spectra (Figure 6) were overlaid according to increasing DMSO content. Monitoring the f_F_ folded protein fraction by ^1^H-^15^N HSQC spectra as a function of DMSO concentration, the unfolding enthalpy ∆*G*_F-U_ can be determined from the following equations [38]:

Δ*G*_F-U_ = Δ*G*^0^ _F-U_ − m [DMSO] (1)

f_F_ = (1+ exp((−Δ*G*^0^ _F-U_ + m [DMSO])/RT))^−1^ (2)

Δ*G*^0^ _F-U_ = m [DMSO]_50_ (3)

I/I_0_ = a/(1 + exp(m ([DMSO-[DMSO]_50_)/RT) (4)

The experimental I/I_0_ ratios are fitted by m (sensitivity of unfolding) and the [DMSO]_50_ concentration, where half of the protein is unfolded.

According to NMR, the native protein started to unfold at 40% DMSO concentration, and all signals disappeared at ca. 82% DMSO content, showing the complete loss of the secondary structure. However, due to the intact S-S bonds, the DMSO-induced unfolding of PAF was reversible around room temperature. 

**Figure 7:**
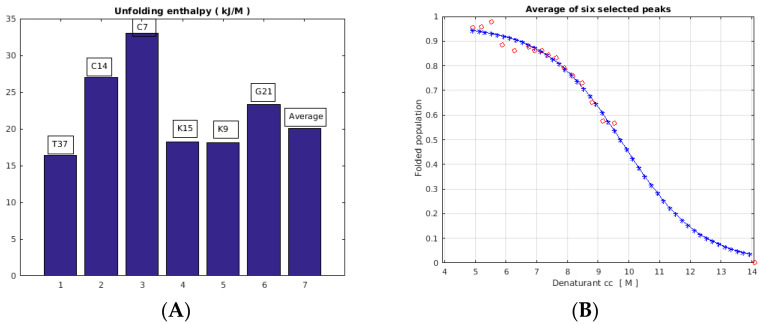
The NMR unfolding enthalpies as derived for six well-separated peaks of PAF (C7, K9, C14, K15, G21, and T37) and for the average (**A**), as obtained from HSQC experiments measured at 310 K temperature. For the average of six peak volumes (**B**), we obtained ∆G_F-U_ = 20.0 kJ/M, and the fit error was 3.1%, while the individual errors were in the 4–9% range (see also Figure S3).

The analysis of the NMR unfolding experiments (Figure 7) yielded ∆G_F-U_ = 23 ± 7 kJ/M as the enthalpy for the average of six separate peaks, while the individual values were scattered in the 17–33 kJ/M range. The positive value indicates an endothermic process. Evaluation of two more peaks (T4 and D55) yielded values of 11 and 18 kJ/M, respectively (13 and 10% fit errors, not shown), suggesting that residues closer to termini are in the low-enthalpy range. The changes in the ^1^H/^15^N chemical shifts upon DMSO titration could be the consequence of conformational changes due to increasing DMSO content and/or fast chemical exchange on the NMR timescale with the invisible unfolded component.

## 3. Discussion

To obtain more insight about the unfolding process, the three-dimensional structures of both PAF and PAF^D19S^ were determined in a 50% DMSO/water solvent mixture. There must be a significant unfolded protein fraction in this environment; however, they are invisible to NMR. Thus, we were able to study only the folded fraction of the proteins. We found that their 3D structures were mostly conserved after adding 50% DMSO to the solution. Still, their β1- and β5-strands were slightly different and more flexible due to the impact of DMSO. The local backbone dynamics of PAF and PAF^D19S^ showed a very similar picture, and increased mobility was mostly detected near their N- and C-termini. Interestingly, the sporadic (<1%, CEST) conformers found in the water solution of PAF predicted that the unfolding may start close to the PAF termini [28]. In contrast, the majority of side chains of both proteins proved to be quite flexible in the structural ensemble, showing decreased stability in 50% DMSO. This also means that the side-chain H-bond network, described previously for both proteins [16,25,28,39], might be less stable and probably partially disrupted [40], since DMSO works as a strong H-bond acceptor. Furthermore, the disulfide bonds are significantly closer to one another, thereby forming smaller hydrophobic cores.

We increased the temperatures in the NMR experiments in order to shift the global correlation times to faster regions, to provide a better comparison with earlier PAF structures in aqueous solutions. We found that the PAF structures in the mixed solvents were insensitive to a 12 K temperature change. Therefore, we can assume that the structure of PAF^D19S^ is also retained in the slower motional regime.

Interestingly the three-dimensional structure of PAF^D19S^ in 50% DMSO (7NXI) was more similar to that of native PAF (2MHV) than to its own conformation (2NB0) in water. The possible reasons for this are threefold: First, PAF^D19S^ may form slightly different H-bond patterns due to the replacement of the acidic Asp residue with a neutral Ser. It seems that in a less polar environment this becomes less important, so the mutant more closely resembles a PAF structure. Second, as mentioned above, the side chains—including Ser19—are much more flexible in mixed solvents. Third, in previous thermal unfolding experiments [28] we found that the mutant is a two-state folder, in contrast to the native PAF. In this study, the DMSO-induced unfolding (note that this is different from thermal unfolding) could be fitted by two-state models both by NMR and DSC. Thus, the conformational differences between the native PAF and the mutant may be less significant in the presence of DMSO.

The DSC monitoring of DMSO-induced unfolding proved the expected stability loss of both PAF and the inactive mutant, as reflected in the gradual decrease in the protein melting points with increasing DMSO concentration.

Both the ^1^H/^15^N chemical shifts and the peak volumes of the NMR-visible protein fraction of PAF changed upon controlling the DMSO concentration at 310 K (Figure 6, Figures S1 and S2). Some well-resolved NMR peak volumes were successfully fitted with a two-state model, and the unfolding enthalpy could be determined for some residues (Figure 7). We can attribute the chemical shift changes both to a possible conformational drift and to a contribution from the low-populated unfolded fraction(s) in fast exchange with the folded fraction. A putative explanation of the chemical shift drift (Figure 6) caused by the increasing unfolded protein population may be that at least two ”unfolded” conformers were in slow exchange with one another while they were in fast exchange with the folded conformer. 

## 4. Materials and Methods

### 4.1. Differential Scanning Calorimetry (DSC)

PAF and PAF^D19S^ samples were initially dissolved in acetate buffer (20 mM acetic acid, pH = 4.5)/DMSO mixtures. The protein concentration was 100 and 37.5 µM for PAF and 56 and 37.5 µM for PAF^D19S^, respectively, and it remained constant for all samples. Measurements were carried out at 0, 5, 10, 15, 20, 25, 30, 35, 40, 45, 50, 55, and 62.5% DMSO contents. A Malvern MicroCal PEAQ-DSC automatic microcalorimeter was used for all experiments in the temperature range 40–130 °C, with a scan rate of 200 °C/h. For all measurements, three reference buffers and a sample scan were collected. The data were analyzed by subtracting the thermogram of the reference buffer as well as the baseline using the software package supplied with the instrument. The reversibility of the thermal transition of the proteins was also proven by running a second scan after cooling down from the first one. The observed T_m_ melting temperatures were reproducible within 1.0 °C in parallel DSC experiments for all samples. 

### 4.2. NMR Spectroscopy: Signal Assignments, Structure Calculations, and DMSO Titration

Unlabeled and ^15^N-isotope-labelled PAF and PAF^D19S^ were overexpressed and purified using the standard protocol [41] in *Penicillium chrysogenum*. The general methods for the NMR investigation of PAF and PAF^D19S^ were nearly identical; 50% DMSO-d_6_- 50% acetate buffer (20 mM acetic acid, pH = 4.5, 5% D_2_O) solvent mixtures were used for the NMR experiments, where the concentration of both proteins was 1.7 mM. NMR experiments were recorded using NEO or AVANCE-II spectrometers (Bruker) at 700 or 500 MHz proton frequency, at 310 K for PAF and at 298 K for PAF^D19S^. The ^1^H chemical shifts were referenced directly to DSS (2,2-dimethyl-2-sila-pentane-5-sulfonic acid), and indirect ^15^N and ^13^C chemical shift referencing was performed by calculating from the gyromagnetic ratios. Spectra were processed with TopSpin 3.1 (Bruker), and the resonances were assigned with the CCPNmr Analysis 2.5.2 software [42] package. Both sequential and side-chain signals were identified with the help of 2D ^1^H–^1^H TOCSY (80 ms), 2D ^1^H–^1^H NOESY (80 ms), 3D ^15^N-HSQC-TOCSY (70 ms), and 3D ^15^N-HSQC-NOESY (PAF^D19S^ 80ms, PAF 130 ms) spectra. The NOESY spectra described above were used for the structural calculations as well. Disulfide bonds were defined as covalent bond restraints during all structure calculations. The NOE peak assignment and the protein structure determination were accomplished with UNIO’10 [43]. This software package included the CYANA 2.1 version algorithm [44] in our case. The final structures were calculated from 956 (PAF) and 958 (PAF^D19S^) NOEs, respectively. The final structural ensembles included 20 structures with the lowest energies of the calculated 100. 

To study the DMSO-induced unfolding of the proteins, NMR titrations were carried out at 310 K. For this purpose, a series of ^15^N-^1^H HSQC NMR spectra (500.13/50.68 MHz) were recorded with either decreasing or increasing amounts of DMSO-d_6_. The raw integrals of six well-separated cross-peaks were determined using the line shape fit option of TopSpin. However, the viscosity of the solutions changed during the titrations, which also affected the peak volumes, due to the changes in the pertinent ^1^H-T_2_ values. Therefore, at each titration point, two HSQC experiments were performed. First was a fast-HSQC (Bruker pulse sequence: fhsqcf3gpph) experiment [45], followed by a second, modified sequence where extra spin-echo and an additional watergate sequence were inserted at the beginning and end of the original pulse sequence (see Figure S5 for details). This modification doubled the time (5.5 ms vs. 11 ms) of the proton magnetization spent in the transversal plane. Assuming an exponential T_2_ decay, the initial proton magnetization at zero time could be back-calculated from the two experimental points by linear extrapolation, which was used as a correction. To this end, an in-house written MATLAB program was used where, in addition to relaxation correction, the solution volume change (estimated from the height of the solution in the NMR tube) and ^1^H pulse calibration (small changes) were also considered. The starting DMSO-d_6_ concentrations were 88 and 0%, respectively. The ^15^N-^1^H chemical shift and peak integral changes were analyzed in TopSpin 3.1 (Bruker, Billerica, MA, USA). Then, 1.08 mg of ^15^N-labelled PAF was dissolved in 50 ul uL of acetate buffer (pH 4.5) with an additional 350 μL of DMSO-d_6_ that was titrated (Figure S2) with 350 uL of acetate buffer containing 1.07 mg of ^15^N-PAF to make sure that the protein concentration (0.43 mM) was nearly constant during the titration. In the reversed-direction titration, 0.7 mg of PAF was dissolved in 350 uL of 20 mM acetate buffer (5% D_2_O, pH 4.5). Then, it was titrated with neat DMSO-d_6_ aliquots up to 990 uL that resulted in a final 74 *v/v*% DMSO at the end and still yielded observable HSQC signals. The pH of the solvent mixtures was found to be constant during both titrations. 

NMR relaxation data were acquired from the conventional ^15^N (70.966 or 50.684 MHz) relaxation experiments (T_1_, T_2_, ^15^N-^1^H NOE) for PAF and PAF^D19S^. The standard Bruker pulse programs were used, and they were recorded at the same temperature and in the same buffer composition described earlier. The data analysis was based on the Lipari–Szabo method, implemented in the Bruker Dynamics Center 2.4.8 software package. The M2 model (spherical shape and one global correlation time) of the Lipari–Szabo method [32] was chosen to determine the global correlation time for the proteins and the S^2^ order parameters for each residue. The relaxation data were also analyzed with a reduced spectral density mapping approach using in-house written MATLAB codes.

## 5. Conclusions

The NMR-visible structures of the antifungal disulfide protein PAF and its inactive mutant PAF^D19S^ showed essentially the same rigid β-barrel folds in 50% DMSO/water mixed solvent at 310 and 298 K temperatures, respectively. However, slightly increased backbone flexibility around the N- and C-terminals, as well as at numerous side chains, indicated lower stability and provided some insight into the unfolding process. In contrast, we obtained no structural information about the unfolded fractions of the proteins that were present in thermal equilibrium as a 10–15% population in the presence of 50% DMSO. Still, the NMR and DSC experiments as a function of DMSO concentration yielded useful data on the thermodynamics of DMSO-induced chemical unfolding. The recent data support the reduced stability of the proteins with increasing DMSO concentrations, as reflected in the significant melting temperature reductions. The unfolding was endothermic, with enthalpy ∆G_F-U_ = 23 ± 7 kJ/M, as measured by NMR at a constant temperature of 310 K.

## Figures and Tables

**Table 1:** The parabolic fitting of melting points as T_m_(x) = p_1_*x^2^ + p_2_*x + p_3_ parameters of the DSC experimental data for PAF and PAF^D19S^. The melting points are given as the values from the fits.

Protein	p_1_ (* 10^−3^)	p_2_ (* 10^−3^)	T_m_ (°C) (p_3_) (0% DMSO)	T_m_ (°C) (50% DMSO)	R^2^
PAF	−2.98	−59.0	93.2 ± 0.8	82.8 ± 0.7	0.99
PAF^D19S^	−2.16	−72.2	90.7 ± 1.0	81.7 ± 0.9	0.98

## Data Availability

The protein structures are available from https://www.rcsb.org/. The three-dimensional structures of PAF and PAF^D19S^ in 50% DMSO are available in the PDB database (7PGD and 7NXI, respectively). The NMR chemical shift assignments of these proteins were uploaded to the BMRB (https://bmrb.pdbj.org/) under entries 34655 and 34612.

## Supplementary Materials

The following supporting information can be downloaded at: https://www.mdpi.com/article/10.3390/ijms24021208/s1.
