# Supplementary material for: DMSO-Induced Unfolding of the Antifungal Disulfide Protein PAF and Its Inactive Variant: A Combined NMR and DSC Study
Source: Int J Mol Sci. 2023 Jan 7;24(2):1208. doi: 10.3390/ijms24021208 (PMC9864379; doi:10.3390/ijms24021208)
Supplement: Supplementary file 1 [file ijms-24-01208-s001.zip › ijms-2100032-supplementary.pdf]

Supplementary information:

DMSO Induced Unfolding of the Antifungal Disulfide Protein PAF and its Inactive Variant: a combined NMR and DSC study

András Czajlik [1,2], Ágnes Batta [3], Kinga Kerner [4], Ádám Fizil [5], Dorottya Hajdu [6], Mária Raics [7] Katalin E. Kövér [8] and Gyula Batta [9,*]

Supplementary figures:

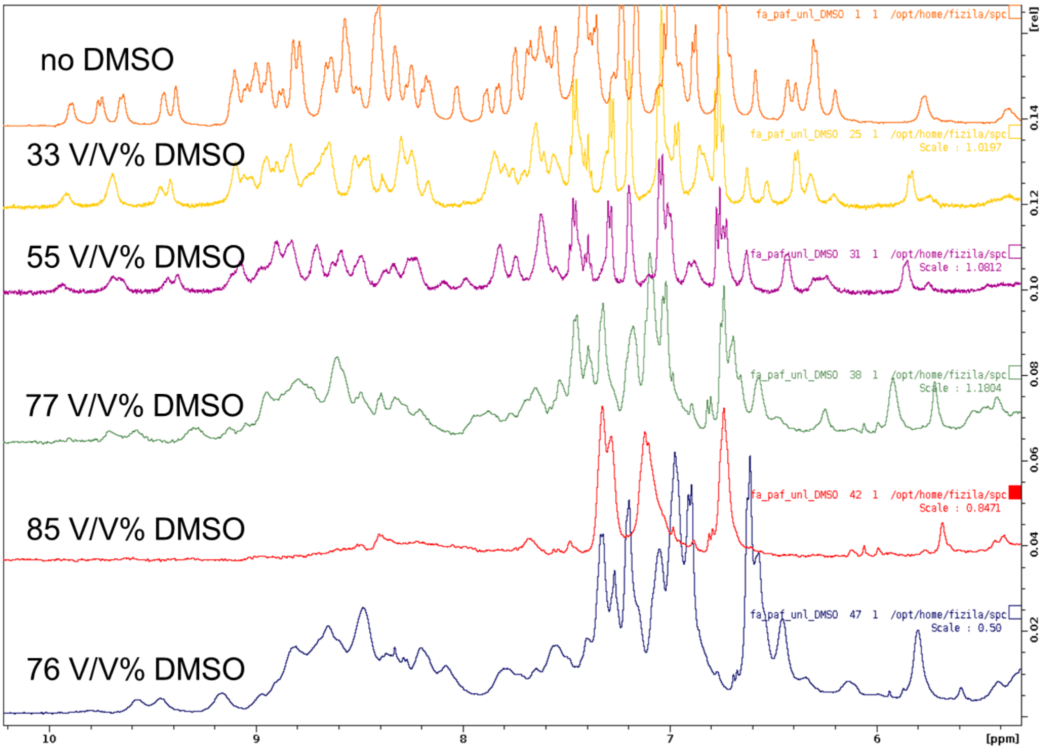

Figure S1 A

DMSO titration of unlabelled PAF as followed ^{1}H NMR spectra at 500.13 MHz. The folded structure disappears at 85 v/v % DMSO, but dilution again with the buffer shows that refolding starts and unfolding is reversible (spectrum at the bottom, at 76%).

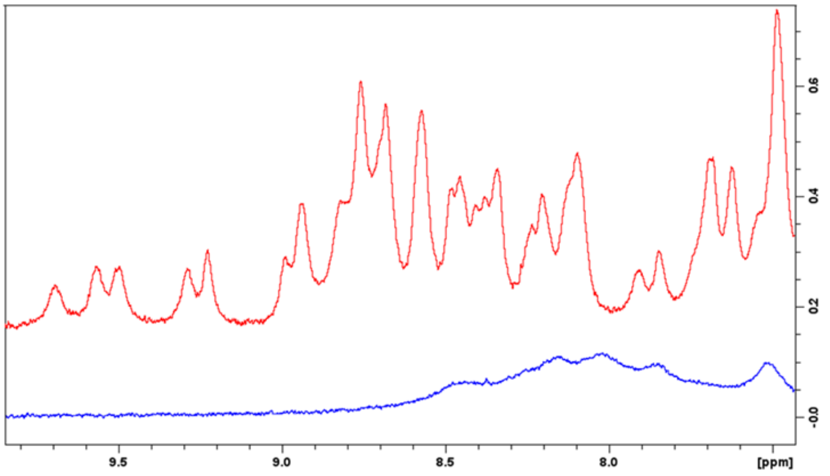

Figure S1 B

Unlabelled PAF dissolved in 93 v/v % DMSO-d_6 at 300K as detected by 500.13 MHz ^{1}H NMR (blue). Dilution with aqueous acetate buffer shows that folded structures are present again at 50% v/v % DMSO, as shown by the pop up of NH signals (red).

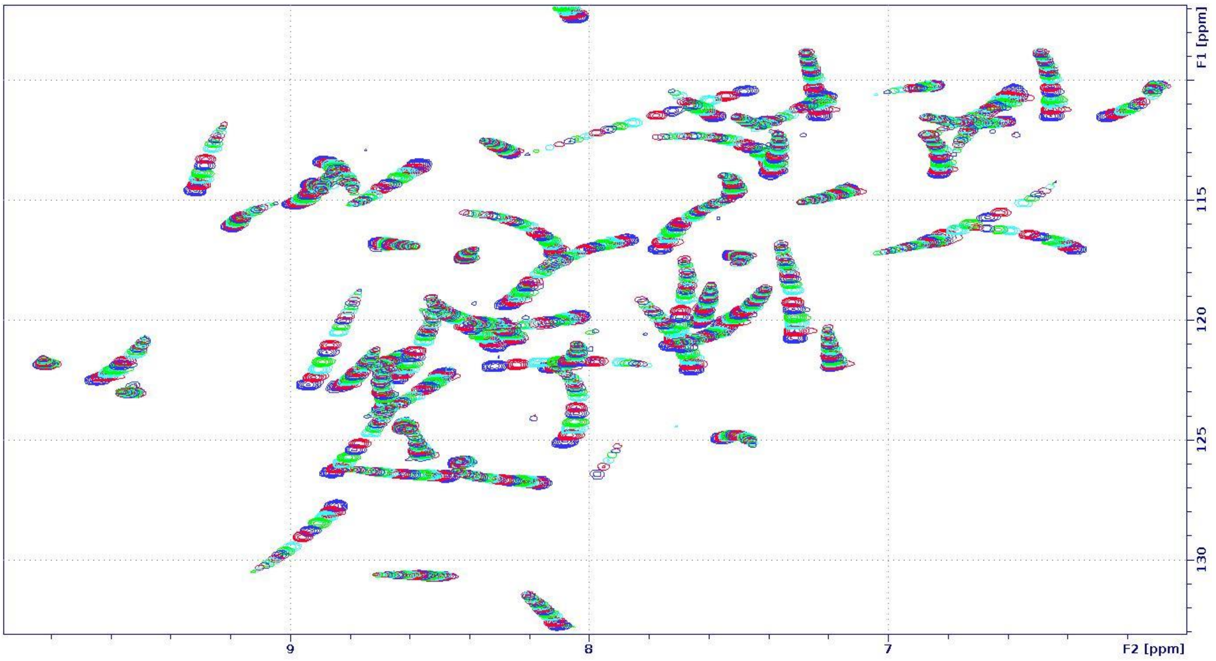

Figure S2

The serial of ^{1}H-^{15}N HSQC spectra (310K) using a Bruker AVANCE-II 500 MHz spectrometer shows the gradual increase of peak intensities and shift of peak positions upon dilution with aqueous buffer. Protein concentration was kept constant during the titration as described in the experimental part. The experiment was started at 10M DMSO-d_6 and the strongest blue peaks belong to the lowest DMSO concentration.

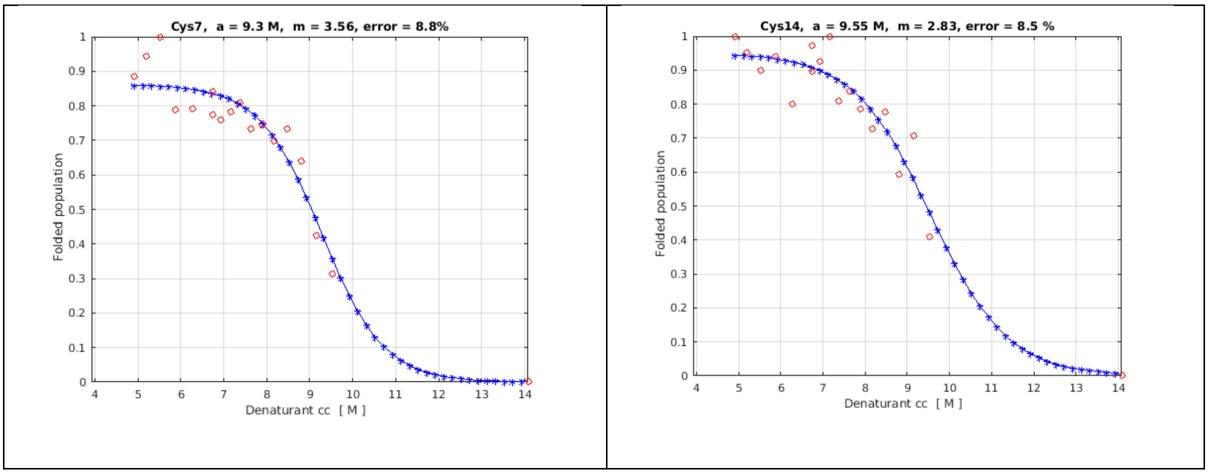

Figure S3

Fitting the DMSO induced unfolding according to peak volume intensity changes of Cys7 and Cys14, as a function of denaturant concentration.

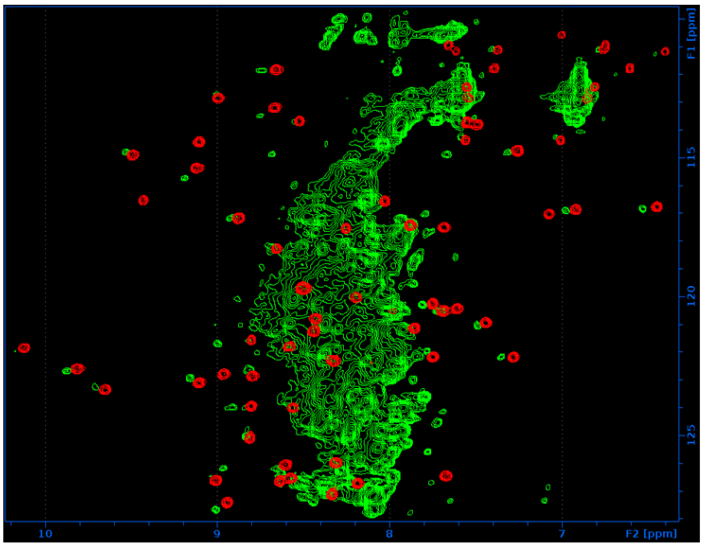

Figure S4

Irreversible heat denaturation of PAF using dithiothreitol (DTT) as reducing agent and incubated at 363K for 1 hr. Overlayed ^{15}N HSQC NMR spectra of native (red) and denatured (green) PAF as compared later at 310K temperature. Inactive oligomers were formed up to tetramers as found also by MS and NMR DOSY experiments.

Quantitative 2D ^{1}H-^{15}N HSQC

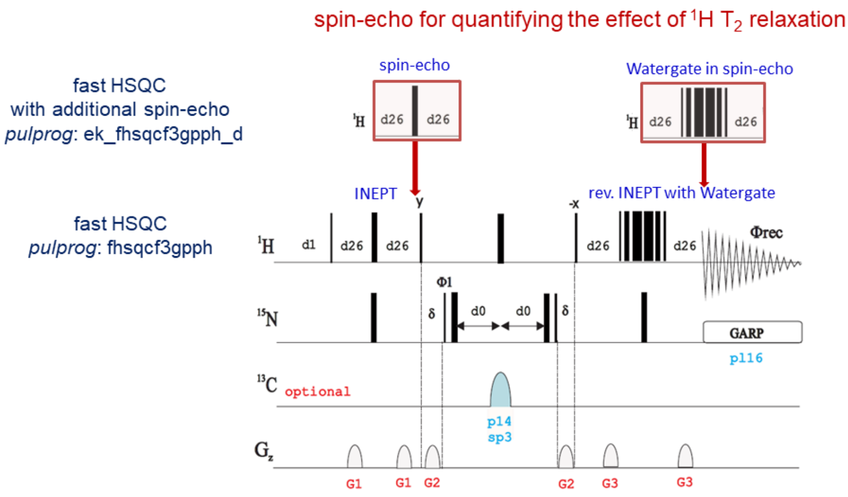

Figure S5

Scheme and pulse program pair for relaxation compensated ^{1}H/^{15}N HSQC experiments that allows quantitative evaluation of HSQC experiments. Additional spin-echo sequences with refocusing of ^{1}H chemical shift and $^1J_{NH}$ evoultion and matching the duration of INEPT/reverse INEPT blocks are included for quantifying the signal intensity loss due to ^{1}H T_2 relaxation.

```
;Pulse program 1 (Bruker)
;fhsqcf3gpph
;avance-version (09/07/23)
;2D H-1/X correlation via double inept transfer
;phase sensitive
;with decoupling during acquisition
;S. Mori, C. Abeygunawardana, M. O'Neil-Johnson & P.C.M. van Zijl,
;   J. Magn. Reson. B 108, 94-98 (1995)
;$CLASS=HighRes
;$DIM=2D
;$TYPE=
;$SUBTYPE=
;$COMMENT=

#include <Avance.incl>
#include <Grad.incl>
#include <Delay.incl>

"p2=p1*2"
"p22=p21*2"
"d11=30m"
"d12=20u"
"d13=4u"
"d26=1s/(cnst4*4)"
"in0=inf1/2"
"d0=3u"
"DELTA=d19-p22/2"
"DELTA1=d26-p16-d16-p27*3-d19*5-p1*2/PI"
"DELTA2=d26-p16-d16-p27*2-p0-d19*5-de-8u"
```

```
"DELTA4=p21*2/3.1416"

#   ifdef LABEL_CN
"DELTA3=d0+larger(p2,p14)/2"
#   else
"DELTA3=d0+p2/2"
#   endif /*LABEL_CN*/

"TAU=d26-p16-4u"
"acqt0=0"
baseopt_echo

1 ze
  d11 pl16:f3
2 d1 do:f3
3 d12 pl1:f1
  50u UNBLKGRAD
  (p1 ph1)
  4u
  p16:gp1
  TAU pl3:f3
  (center (p2 ph1) (p22 ph6):f3 )
  4u
  p16:gp1
  TAU
  (p1 ph2)
  4u
  p16:gp2
  d16
  (p21 ph3):f3
  DELTA3
  (p22 ph3):f3
  DELTA4
  d0

#   ifdef LABEL_CN
  (center (p2 ph5) (p14:sp3 ph1):f2 )
#   else
  (p2 ph5)
#   endif /*LABEL_CN*/
  d0
  DELTA4
  (p22 ph4):f3
  DELTA3
  (p21 ph4):f3
  4u
  p16:gp2
  d16
  (p1 ph7)
  DELTA1
  p16:gp3
  d16 pl18:f1
  p27*0.231 ph8
  d19*2
  p27*0.692 ph8
  d19*2
  p27*1.462 ph8
  DELTA
  (p22 ph1):f3
  DELTA
  p27*1.462 ph9
  d19*2
  p27*0.692 ph9
```

```
  d19*2
  p0*0.231 ph9
  4u
  p16:gp3
  d16
  4u BLKGRAD
  DELTA2 pl16:f3
  go=2 ph31 cpd3:f3
  d1 do:f3 mc #0 to 2 F1PH(calph(ph3, +90) & calph(ph6, +90), caldel(d0, +in0))
exit

ph1=0
ph2=1
ph3=0 2
ph4=0 0 0 0 2 2 2 2
ph5=0 0 2 2
ph6=0
ph7=2
ph8=1
ph9=3
ph31=0 2 0 2 2 0 2 0


;pl1 : f1 channel - power level for pulse (default)
;pl3 : f3 channel - power level for pulse (default)
;pl16: f3 channel - power level for CPD/BB decoupling
;pl18: f1 channel - power level for 3-9-19-pulse (watergate)
;sp3: f2 channel - shaped pulse 180 degree (adiabatic)
;p0 : f1 channel -  90 degree pulse at pl18
;        use for fine adjustment
;p1 : f1 channel -  90 degree high power pulse
;p2 : f1 channel - 180 degree high power pulse
;p14: f2 channel - 180 degree shaped pulse for inversion (adiabatic)
;p16: homospoil/gradient pulse
;p21: f3 channel -  90 degree high power pulse
;p22: f3 channel - 180 degree high power pulse
;p27: f1 channel -  90 degree pulse at pl18
;d0 : incremented delay (2D)                    [3 usec]
;d1 : relaxation delay; 1-5 * T1
;d11: delay for disk I/O                        [30 msec]
;d12: delay for power switching                 [20 usec]
;d13: short delay                               [4 usec]
;d16: delay for homospoil/gradient recovery
;d19: delay for binomial water suppression
;     d19 = (1/(2*d)), d = distance of next null (in Hz)
;d26 : 1/(4J(YH))
;cnst4: = J(YH)
;inf1: 1/SW(X) = 2 * DW(X)
;in0: 1/(2 * SW(X)) = DW(X)
;nd0: 2
;NS: 8 * n
;DS: 16
;td1: number of experiments
;FnMODE: States-TPPI (or TPPI)
;cpd3: decoupling according to sequence defined by cpdprg3
;pcpd3: f3 channel - 90 degree pulse for decoupling sequence

;use gradient ratio:         gp 1 : gp 2 : gp 3
;                              50 :   80 :   30

;for z-only gradients:
;gpz1: 50%
;gpz2: 80%
```

```
;gpz3: 30%

;for magic angle gradients (MAG) with xyz-gradientsystem use instead:
;gpx1: 20%
;gpx2: 10%
;gpx3: 15%*k
;gpy1: 40%
;gpy2: 0%
;gpy3: 15%*k
;gpz1: 20%
;gpz2: 0%
;gpz3: 15%

;   with k = calibration constant from 1D magic angle gradient
;      calibration

;use gradient files:
;gpnam1: SMSQ10.100
;gpnam2: SMSQ10.100
;gpnam3: SMSQ10.100

  ;preprocessor-flags-start
;LABEL_CN: for C-13 and N-15 labeled samples start experiment with
;            option -DLABEL_CN (eda: ZGOPTNS)
                                    ;preprocessor-flags-end



;$Id: fhsqcf3gpph,v 1.7.2.1 2009/10/29 19:56:58 ber Exp $
```

;Pulse program 2 (pulse program allowing doubled time for relaxation during the pulse sequence)
;ek_fhsqcf3gpph_d

```
;avance-version (07/04/04)
;2D H-1/X correlation via double inept transfer
;phase sensitive
;with decoupling during acquisition

;S. Mori, C. Abeygunawardana, M. O'Neil-Johnson & P.C.M. van Zijl,
;   J. Magn. Reson. B 108, 94-98 (1995)

;based on fhsqcf3gpph for quantitative HSQC - for protein concentration measurement
;double 'INEPT' for compensation of relaxation loss during INEPT-delay
;pair of experiments (ek_fhsqcf3gpph)required for quantification!!
;single+double INEPT!! p16 should be short, ca. 500-600 us

;February 13, 2016 EK, RM,

;$CLASS=HighRes
;$DIM=2D
;$TYPE=
;$SUBTYPE=
;$COMMENT=

#include <Avance.incl>
#include <Grad.incl>
#include <Delay.incl>

"p2=p1*2"
"p22=p21*2"
```

```
"d11=30m"
"d12=20u"
"d13=4u"
"d26=1s/(cnst4*4)"
"d25=(p22-p2)/2"     ;compensate for duration of p22
"in0=inf1/2"
"d0=3u"
"DELTA=d19-p22/2"
"DELTA1=d26-p16-d16-p27*3-d19*5"
"DELTA2=d26-p16-d16-p27*2-p0-d19*5-8u"
"DELTA4=p21*2/3.1416"

#   ifdef LABEL_CN
"DELTA3=d0+larger(p2,p14)/2"
#   else
"DELTA3=d0+p2/2"
#   endif /*LABEL_CN*/

"TAU=d26-p16-4u"

1 ze
  d11 pl16:f3
2 d1 do:f3
3 d12 pl1:f1
  50u UNBLKGRAD
  (p1 ph1)
  4u
  p16:gp1
  TAU pl3:f3
  (center (p2 ph1) (p22 ph6):f3 )  ;INEPT to generate antiphase
  4u
  p16:gp1
  TAU

  d25
  4u
  p16:gp1
  TAU pl3:f3
  (p2 ph1)             ;INEPT to retain antiphase
  4u                   ;to estimate relaxation loss
  p16:gp1
  TAU
  d25

  (p1 ph2)
  4u
  p16:gp2
  d16
  (p21 ph3):f3
  DELTA3
  (p22 ph3):f3
  DELTA4
  d0

#   ifdef LABEL_CN
  (center (p2 ph5) (p14:sp3 ph1):f2 )
#   else
  (p2 ph5)
#   endif /*LABEL_CN*/

  d0
  DELTA4
  (p22 ph4):f3
```

```
  DELTA3
  (p21 ph4):f3
  4u
  p16:gp2
  d16
  (p1 ph7)                    ;for double INEPT experiment

  DELTA1
  p16:gp3                     ;generate in-phase
  d16 pl18:f1
  p27*0.231 ph8
  d19*2
  p27*0.692 ph8
  d19*2
  p27*1.462 ph8
  DELTA
  (p22 ph1):f3
  DELTA
  p27*1.462 ph9
  d19*2
  p27*0.692 ph9
  d19*2
  p0*0.231 ph9
  4u
  p16:gp3
  d16
  4u
  DELTA2

  DELTA1                      ;retain in-phase
  p16:gp3
  d16 pl18:f1
  p27*0.231 ph8
  d19*2
  p27*0.692 ph8
  d19*2
  p27*1.462 ph8
  d19
  d19
  p27*1.462 ph9
  d19*2
  p27*0.692 ph9
  d19*2
  p0*0.231 ph9
  4u
  p16:gp3
  d16
  4u BLKGRAD
  DELTA2 pl16:f3

  go=2 ph31 cpd3:f3
  d1 do:f3 mc #0 to 2 F1PH(ip3 & ip6, id0)
exit


ph1=0
ph2=1
ph3=0 2
ph4=0 0 0 0 2 2 2 2
ph5=0 0 2 2
ph6=0
ph7=0
ph8=1
```

ph9=3
ph31=0 2 0 2 2 0 2 0

;pl1 : f1 channel - power level for pulse (default)
;pl3 : f3 channel - power level for pulse (default)
;pl16: f3 channel - power level for CPD/BB decoupling
;pl18: f1 channel - power level for 3-9-19-pulse (watergate)
;sp3: f2 channel - shaped pulse 180 degree (adiabatic)
;p0 : f1 channel - 90 degree pulse at pl18
; use for fine adjustment
;p1 : f1 channel - 90 degree high power pulse
;p2 : f1 channel - 180 degree high power pulse
;p14: f2 channel - 180 degree shaped pulse for inversion (adiabatic)
;p16: homospoil/gradient pulse
;p21: f3 channel - 90 degree high power pulse
;p22: f3 channel - 180 degree high power pulse
;p27: f1 channel - 90 degree pulse at pl18
;d0 : incremented delay (2D) [3 usec]
;d1 : relaxation delay; 1-5 * T1
;d11: delay for disk I/O [30 msec]
;d12: delay for power switching [20 usec]
;d13: short delay [4 usec]
;d16: delay for homospoil/gradient recovery
;d19: delay for binomial water suppression
; d19 = (1/(2*d)), d = distance of next null (in Hz)
;d26 : 1/(4J(YH))
;cnst4: = J(YH)
;inf1: 1/SW(X) = 2 * DW(X)
;in0: 1/(2 * SW(X)) = DW(X)
;nd0: 2
;NS: 8 * n
;DS: 16
;td1: number of experiments
;FnMODE: States-TPPI (or TPPI)
;cpd3: decoupling according to sequence defined by cpdprg3
;pcpd3: f3 channel - 90 degree pulse for decoupling sequence

;use gradient ratio: gp 1 : gp 2 : gp 3
; 50 : 80 : 30
;for z-only gradients:
;gpz1: 50%
;gpz2: 80%
;gpz3: 30%

;for magic angle gradients (MAG) with xyz-gradientsystem use instead:

;gpx1: 20%
;gpx2: 10%
;gpx3: 15%*k

;gpy1: 40%
;gpy2: 0%
;gpy3: 15%*k

;gpz1: 20%
;gpz2: 0%
;gpz3: 15%

; with k = calibration constant from 1D magic angle gradient
; calibration

```
;use gradient files:
;gpnam1: SINE.100
;gpnam2: SINE.100
;gpnam3: SINE.100

                                    ;preprocessor-flags-start
;LABEL_CN: for C-13 and N-15 labeled samples start experiment with
;            option -DLABEL_CN (eda: ZGOPTNS)
                                    ;preprocessor-flags-end


;$Id: fhsqcf3gpph,v 1.6 2007/04/11 13:34:29 ber Exp $
```

Table S1

^{15}N NMR relaxation data as measured for PAF at 50 v/v% DMSO-d_6 concentration at 310K temperature using a Bruker NEO/Avance III 700 MHz spectrometer. For data evaluation the manufacturers „Dynamics Center" software package was used with the M2 model of the relaxation module. The global correlation time derived from these data was τ_c = 7.7 ± 0.3 ns.

name	NOE	error	T1[s]	error	T2[s]	error	S2(M2)	error
2Lys	0,672	0,017	0,862	0,091	0,110	0,004	0.745	0.0117
3Tyr	0,761	0,008	0,826	0,055	0,100	0,003	0.823	0.00921
4Thr	0,735	0,007	0,900	0,058	0,095	0,001	0.857	0.00466
5Gly	0,751	0,004	0,823	0,053	0,103	0,001	0.792	0.00459
6Lys	0,802	0,006	0,809	0,050	0,088	0,002	0.923	0.00734
7Cys	0,847	0,008	0,751	0,038	0,089	0,001	0.919	0.00564
8Thr	0,797	0,010	0,804	0,056	0,087	0,002	0.929	0.00791
9Lys	0,803	0,010	0,789	0,050	0,067	0,003	0.956	0.00580
10Ser	0,743	0,013	0,785	0,064	0,092	0,004	0.885	0.0141
11Lys	0,704	0,012	0,839	0,074	0,105	0,002	0.775	0.00576
12Asn	0,786	0,008	0,817	0,061	0,078	0,002	0.948	0.00458
13Glu	0,803	0,007	0,806	0,045	0,092	0,001	0.889	0.00519
14Cys	0,812	0,010	0,787	0,054	0,093	0,001	0.876	0.00513
15Lys	0,810	0,009	0,786	0,048	0,083	0,004	0.943	0.0146
16Tyr	0,853	0,008	0,727	0,047	0,094	0,002	0.853	0.0123
17Lys	0,745	0,007	0,833	0,039	0,101	0,001	0.807	0.00392
18Asn	0,714	0,007	0,815	0,041	0,098	0,002	0.834	0.00609
19Asp	0,666	0,006	0,794	0,061	0,105	0,002	0.778	0.00672
20Ala	0,708	0,005	0,861	0,046	0,099	0,002	0.820	0.00608
21Gly	0,749	0,006	0,803	0,039	0,095	0,002	0.863	0.00879
22Lys	0,707	0,005	0,782	0,033	0,091	0,002	0.890	0.00611
23Asp	0,723	0,005	0,848	0,044	0,095	0,005	0.840	0.0142
24Thr	0,733	0,006	0,813	0,047	0,099	0,001	0.823	0.00452
25Phe	0,780	0,006	0,819	0,045	0,100	0,002	0.823	0.00661
26Ile	0,791	0,008	0,798	0,035	0,093	0,001	0.875	0.00418
27Lys	0,764	0,006	0,826	0,024	0,084	0,003	0.903	0.00817
28Cys	0,805	0,011	0,826	0,037	0,087	0,002	0.913	0.00971
30Lys	0,686	0,007	0,843	0,036	0,091	0,001	0.876	0.00530
31Phe	0,428	0,007	0,828	0,047	0,106	0,002	0.759	0.00422
32Asp	0,698	0,007	0,771	0,069	0,094	0,004	0.855	0.0192
33Asn	0,671	0,006	0,775	0,056	0,088	0,002	0.882	0.00523
34Lys	0,725	0,010	0,775	0,037	0,080	0,002	0.929	0.00536
35Lys	0,729	0,007	0,761	0,038	0,098	0,002	0.813	0.0104
36Cys	0,805	0,008	0,776	0,045	0,089	0,001	0.920	0.00500
37Thr	0,785	0,016	0,797	0,085	0,071	0,002	0.986	0.00920
38Lys	0,790	0,007	0,838	0,059	0,079	0,002	0.948	0.00351
39Asp	0,745	0,006	0,873	0,027	0,079	0,002	0.919	0.00568
40Asn	0,788	0,010	0,825	0,042	0,097	0,002	0.849	0.00829
41Asn	0,823	0,006	0,769	0,039	0,076	0,002	0.965	0.00402
43Cys	0,830	0,009	0,821	0,033	0,083	0,001	0.962	0.00608
44Thr	0,829	0,009	0,837	0,044	0,091	0,003	0.888	0.0101
45Val	0,821	0,009	0,816	0,050	0,095	0,002	0.859	0.00693
46Asp	0,833	0,008	0,826	0,042	0,082	0,004	0.932	0.0138
48Tyr	0,704	0,006	0,702	0,075	0,096	0,003	0.830	0.0130
49Asn	0,693	0,007	0,814	0,038	0,111	0,002	0.746	0.00628
50Asn	0,692	0,006	0,839	0,037	0,102	0,001	0.802	0.00432
51Ala	0,706	0,006	0,822	0,035	0,097	0,002	0.839	0.00579
52Val	0,749	0,008	0,865	0,035	0,106	0,002	0.776	0.00471
53Asp	0,794	0,009	0,836	0,033	0,091	0,001	0.892	0.00595
54Cys	0,753	0,011	0,851	0,045	0,079	0,002	0.938	0.00547
55Asp	0,759	0,010	0,825	0,046	0,069	0,002	0.962	0.00495

^{15}N NMR relaxation data as measured for PAF-D19S at 50 v/v% DMSO-d_6 concentration at 298K temperature using a Bruker Avance II 500 MHz spectrometer. The global correlation time derived from these data (similarly to described at Table S1 A) was τ_c = 12.0 ± 0.3 ns.

Table S1B

name	NOE	error	T1[s]	error	T2[s]	error	T1/T2	error	S2(M2)	error
2Lys	0,528	0,007	0,729	0,057	0,091	0,003	8,013	0,292	0.692	0.00826
4Thr	0,710	0,007	0,818	0,025	0,072	0,001	11,369	0,180	0.835	0.00612
5Gly	0,713	0,004	0,724	0,022	0,077	0,001	9,431	0,137	0.820	0.00468
6Lys	0,779	0,007	0,730	0,016	0,069	0,001	10,605	0,129	0.899	0.00529
7Cys	0,832	0,010	0,732	0,039	0,068	0,001	10,760	0,255	0.912	0.00461
8Thr	0,766	0,010	0,727	0,019	0,069	0,001	10,462	0,141	0.894	0.00570
9Lys	0,804	0,011	0,733	0,067	0,070	0,002	10,441	0,445	0.885	0.0126
10Ser	0,708	0,007	0,713	0,056	0,077	0,001	9,259	0,317	0.805	0.00403
11Lys	0,669	0,009	0,765	0,061	0,081	0,002	9,461	0,350	0.772	0.00927
12Asn	0,752	0,008	0,737	0,035	0,071	0,003	10,385	0,269	0.876	0.0110
13Glu	0,769	0,008	0,752	0,053	0,070	0,003	10,809	0,371	0.886	0.0123
14Cys	0,803	0,013	0,760	0,036	0,071	0,002	10,638	0,267	0.868	0.0104
15Lys	0,790	0,011	0,747	0,041	0,067	0,001	11,180	0,280	0.923	0.00712
16Tyr	0,787	0,008	0,699	0,029	0,072	0,003	9,734	0,243	0.897	0.0111
17Lys	0,648	0,007	0,785	0,033	0,081	0,002	9,739	0,207	0.777	0.00708
18Asn	0,710	0,008	0,725	0,028	0,075	0,001	9,651	0,171	0.831	0.00496
19Ser	0,609	0,008	0,720	0,048	0,083	0,001	8,641	0,259	0.746	0.00489
20Ala	0,659	0,006	0,763	0,035	0,077	0,002	9,965	0,224	0.813	0.00755
21Gly	0,685	0,007	0,740	0,029	0,072	0,005	10,223	0,329	0.863	0.0122
22Lys	0,670	0,006	0,734	0,023	0,074	0,001	9,919	0,148	0.842	0.00493
23Asp	0,641	0,006	0,789	0,028	0,080	0,002	9,835	0,180	0.780	0.00649
24Thr	0,762	0,008	0,734	0,029	0,075	0,001	9,727	0,172	0.823	0.00291
25Phe	0,769	0,008	0,755	0,029	0,075	0,002	10,071	0,215	0.841	0.00871
26Ile	0,754	0,010	0,734	0,025	0,071	0,002	10,300	0,183	0.875	0.00737
27Lys	0,728	0,008	0,756	0,037	0,074	0,002	10,169	0,233	0.836	0.00683
28Cys	0,780	0,013	0,748	0,040	0,066	0,002	11,268	0,309	0.916	0.0113
30Lys	0,683	0,008	0,768	0,027	0,070	0,002	11,009	0,206	0.867	0.00754
31Phe	0,359	0,007	0,777	0,035	0,089	0,002	8,714	0,202	0.705	0.00715
32Asp	0,625	0,006	0,734	0,021	0,077	0,002	9,596	0,153	0.828	0.00644
33Asn	0,617	0,007	0,722	0,017	0,078	0,001	9,223	0,115	0.814	0.00482
34Lys	0,675	0,011	0,711	0,020	0,063	0,001	11,335	0,152	0.940	0.00357
35Lys	0,717	0,009	0,722	0,024	0,074	0,001	9,705	0,161	0.846	0.00615
36Cys	0,793	0,011	0,713	0,022	0,066	0,001	10,761	0,172	0.931	0.00687
37Thr	0,821	0,018	0,744	0,039	0,066	0,002	11,338	0,293	0.932	0.0101
38Lys	0,773	0,007	0,792	0,028	0,065	0,001	12,214	0,200	0.931	0.00534
39Asp	0,723	0,007	0,772	0,043	0,066	0,001	11,604	0,287	0.922	0.00533
40Asn	0,801	0,013	0,739	0,031	0,073	0,002	10,111	0,233	0.863	0.00957
41Asn	0,749	0,005	0,737	0,033	0,069	0,002	10,684	0,240	0.894	0.00888
42Lys	0,712	0,008	0,754	0,028	0,067	0,001	11,295	0,201	0.912	0.00566
43Cys	0,765	0,009	0,775	0,038	0,060	0,002	12,912	0,310	0.951	0.00737
44Thr	0,769	0,010	0,769	0,045	0,069	0,001	11,181	0,287	0.900	0.00336
45Val	0,784	0,011	0,749	0,037	0,071	0,001	10,496	0,243	0.870	0.00681
46Asp	0,776	0,009	0,750	0,025	0,073	0,002	10,240	0,194	0.858	0.00803
47Thr	0,713	0,004	0,724	0,022	0,077	0,001	9,431	0,137	0.820	0.00468
48Tyr	0,672	0,007	0,706	0,051	0,080	0,001	8,884	0,284	0.782	0.00546
49Asn	0,526	0,005	0,712	0,030	0,082	0,006	8,717	0,301	0.828	0.0110
50Asn	0,653	0,007	0,760	0,024	0,077	0,002	9,801	0,161	0.810	0.00627
51Ala	0,663	0,005	0,751	0,032	0,077	0,001	9,736	0,189	0.806	0.00508
52Val	0,702	0,008	0,803	0,018	0,080	0,002	10,065	0,168	0.792	0.00638
53Asp	0,788	0,011	0,761	0,033	0,069	0,003	10,993	0,274	0.881	0.0109
54Cys	0,776	0,012	0,761	0,039	0,060	0,002	12,620	0,334	0.954	0.00967
55Asp	0,714	0,010	0,781	0,045	0,056	0,002	13,896	0,386	0.940	0.00487
